# Synergy of Hydeal-D^®^ and Hyaluronic Acid for Protecting and Restoring Urothelium: In Vitro Characterization

**DOI:** 10.3390/pharmaceutics13091450

**Published:** 2021-09-11

**Authors:** Marco Ruggeri, Mauro Pavan, Matteo Soato, Susi Panfilo, Carlo Barbera, Devis Galesso, Dalila Miele, Silvia Rossi, Alba Di Lucia, Franca Ferrari, Giuseppina Sandri

**Affiliations:** 1 Department of Drug Sciences, University of Pavia, Viale Taramelli 12, 27100 Pavia, Italy; marco.ruggeri02@universitadipavia.it (M.R.); dalila.miele@unipv.it (D.M.); silvia.rossi@unipv.it (S.R.); franca.ferrari@unipv.it (F.F.); 2 Fidia Farmaceutici S.p.A., Via Ponte Della Fabbrica 3/A, 35031 Abano Terme, Italy; mpavan@fidiapharma.it (M.P.); msoato@fidiapharma.it (M.S.); spanfilo@fidiapharma.it (S.P.); CBarbera@fidiapharma.it (C.B.); DGalesso@fidiapharma.it (D.G.)

**Keywords:** HydealCyst, hyaluronic acid, interstitial cystitis, urothelial cell model, mucoadhesion, in vitro inflammatory model, glycosaminoglycans secretion

## Abstract

Interstitial cystitis (IC) or painful bladder syndrome is a chronic dysfunction due to an inflammatory condition, characterized by bladder pain and urinary frequency. Currently, no gold standard therapy is available since IC does not respond to conventional ones. Given these premises, the aim of this work was the in vitro characterization of biological properties (mucoadhesion and anti-inflammatory activity) of a commercial product (HydealCyst–HydC) based on hyaluronic acid (HA) and the benzyl ester of HA (Hydeal-D^®^) intended for bladder instillation to restore and/or protect the urothelial layer of glycosamino glycans (GAGs). The in vitro characterization demonstrated that an interaction product is formed between HA and Hydeal-D^®^ that has a role in the rheological behavior and mucoadhesive properties. HA was identified as a key component to form the mucoadhesive joint, while the interaction of HA with Hydeal-D^®^ improved polysaccharide stability and prolonged the activity *ex vivo*. Moreover, HydC is cytocompatible with urothelial cells (HTB-4) and possesses an anti-inflammatory effect towards these cells by decreasing the secretion of IL-6 and IL-8, which were both increased in patients with IC, and by increasing the secretion of sulfated GAGs. These two findings, along with the resilience properties of the formulation due to mucoadhesion, suggest the active role of HydC in protecting and restoring urothelium homeostasis.

## 1. Introduction

Interstitial cystitis (IC) or painful bladder syndrome is a chronic dysfunction of the pelvic walls, characterized by bladder pain and urinary frequency. It is an inflammatory condition affecting people of any age and gender, and it mostly occurs in women [1]. The progression of this disease is slow but constantly worsening, with the deterioration of bladder functions and a negative impact on the quality of life, leading to psychological disorders, such as anxiety, insomnia, and depression [2,3].

The etiology is multifactorial and is based on numerous hypotheses: urinary tract infection, surgery, or viral illness have been proposed, but the most accredited hypothesis is a progressive weakening of the bladder wall lining, consisting of glycosaminoglycans (GAGs) including hyaluronic acid, chondroitin sulphate, heparan sulphate, and dermatan sulphate. GAGs, in physiological conditions, coat the urothelium and play a crucial role in protecting the bladder and preventing the adhesion of pathogenic bacteria to the bladder [4,5,6]. Following damage to this protective layer, the infiltration of harmful urine components with inflammatory action into the underlying layers may occur, with consequent activation of mast cells [7]. These cells, through migration, multiplication, and degranulation phenomena, release chemical mediators, such as cytokines, vasoactive amines, proteolytic enzymes, bradykinin, neuropeptides, histamine, and serotonin, characterized by pro-inflammatory activity in the urothelium. Mast cell hyper-activation stimulates unmyelinated C fibers, leading to bladder pain, and neuro-peptides release, causing secondary damage to the mucosa and fibrosis of the submucosa [8]. Abnormal GAGs excretion in patients with chronic interstitial cystitis has been reported, and this reflects both an abnormality in GAGs metabolism or a correlation between endogenous urinary GAGs and damaged bladder epithelium [9].

The first-line treatment of interstitial cystitis involves pain relief, psychological support, changes in eating habits, and muscular relaxation of the pelvic floor [10], since interstitial cystitis does not respond to conventional therapy with antibiotics. The association of different treatments seems crucial, and, in some cases, anticholinergic drugs, tricyclic antidepressants, and antihistamines are considered. For the treatment of pain, antispasmodics and anti-inflammatories are used. Moreover, bladder instillations of GAGs have been proposed with the aim to re-build the native GAG layer of the bladder and to restore its protective function [11,12,13].

Hyaluronic acid (HA) is a mucopolysaccharide based on two sugars, D-glucuronic acid and N-acetyl-D-glucosamine, bonded with alternating β-1,4 and β-1,3 glycosidic bonds [14]. It has a high molecular mass and is characterized by interesting viscoelastic and rheological behavior, due to its polymeric and polyelectrolyte properties. In the pharmaceutical field, it is used both as an excipient, as a component of medical devices (i.e., to compensate the insufficiency of synovial fluid in arthritic patients through intra-articular injections) and as an active ingredient [15,16,17]. HA is present in numerous fluids and biological tissues, in which it plays either a structural or a biological role. In fact, it promotes fibroblasts and endothelial cells’ proliferation and enhances the healing of connective tissue. In the urologic field, HA is considered a good candidate for the restoration of the bladder GAG layer since it increases the secretion of enzymes leading to increased GAGs production, and it is capable to alter epithelial permeability through the stimulation of the expression of tight junction proteins; it exerts an anti-inflammatory effect by decreasing immune cell infiltration into the urothelium, and it inhibits bladder mast cell activation [18,19].

Hydeal-D^®^, also referred to as HYAFF11p50, is a sodium hyaluronate derivative obtained by the partial esterification of free carboxyl groups of glucuronic acid with benzylic alcohol. These chemical changes increase lipophilicity and allow a higher resistance to degradation by hyaluronidases compared to HA to be obtained. Furthermore, the slow cleavage of benzyl moieties from the polysaccharide backbone results in a gradual and prolonged release of hyaluronic acid [20,21].

Given these premises, the aim of this work was the characterization of HydealCyst (HydC), a commercial product based on HA and Hydeal-D^®^, intended for bladder instillation and aimed at restoring and/or protecting the urothelial layer of GAGs. In this study, from the mechanical point of view, the rheological and mucoadhesive properties were investigated. Moreover, the biocompatibility, the anti-inflammatory activity, and the capability of the system to stimulate GAGs production were also evaluated.

## 2. Experimental Part

### 2.1. Materials

Hyaluronic acid (HA) sodium salt (HA-Na), benzyl ester of HA (Hydeal-D^®^), and Hydeal Cyst (HydC; containing 1.8% HA and 0.2% Hydeal-D^®^) were provided by Fidia Farmaceutici S.p.A. (Abano Terme, Italy); Ialuril^®^ Prefil (containing 1.6% HA and 2.0% chondroitin sulfate) was acquired from IBSA (Lodi, Italy). Ultrapure water (UPW) was generated using a water treatment apparatus by Sartorius (Monza, Italy). Euxyl^®^ PE 9010 was purchased from Schülke & Mayr GmbH (Norderstedt, Germany), while all other reagents were supplied by Sigma (Milan, Italy), unless differently specified, and used without further purification.

A HydC vehicle was obtained by dissolving 0.75 mL of Euxyl^®^ PE 9010 in water and acidifying to a pH of 5.0–5.5 with 0.1 mL of a 55 mg/mL lactic acid solution. HA and Hydeal-D^®^ solutions were obtained by dissolving 1.8 g of HA or 0.2 g of Hydeal-D^®^ in 100 mL of HydC vehicle. The solutions were sterilized as reported in the HydC patent [22].

### 2.2. Methods

#### 2.2.1. Viscosity and Rheological Synergism

The rheological analyses were carried out by means of a modular compact rheometer (Anton Paar MCR 102), equipped with a cone/plate combination (C60/1: Ø 60 mm; angle 1°) as a measuring system [23,24].

HydC, HA, and Hydeal-D^®^ were subjected to viscosity measurements at 37 °C (60 s as the equilibrium time) by applying increasing shear rates from 10 to 150 s^−1^. In order to understand the contribution of the single substances to the rheological properties of the product, HA and Hydeal-D^®^ solutions were prepared for these measurements with the same concentration (1.8% and 0.2% *w/w*, respectively) and in the same vehicle as in HydC (see Section 2.1).

The rheological synergism was performed using an 8% *w/w* porcine gastric mucin dispersion (Type II, Sigma-Aldrich, Milan, Italy) in Hanks’ Balanced Salt Solution (HBSS) (Sigma Aldrich, Milan, Italy). Before analysis, the samples were maintained at 37 °C for 5 min in a shaking water bath. All the mucoadhesion measurements were carried out at the physiological temperature of 37 °C. HA and Hydeal-D^®^ solutions were prepared at the same concentration and in the same vehicle as in HydC (see Section 2.1). Then, HydC, HA, or Hydeal-D^®^ were mixed 1:1 with the mucin dispersion, and the rheological synergism parameter (Δ*η*) was calculated at 10 and 150 s^−1^ as follows:

(1) $$\Delta \eta =\eta_{mix}-\left(\eta_{S}+\eta_{muc}\right)$$

where *η_mix_* is the apparent viscosity of the sample (s)–mucin (muc) mixture (mix) and *η_S_* and *η_muc_* are the apparent viscosity of the sample (s) and mucin (muc), respectively, each having the same concentration of the mix.

#### 2.2.2. Washability Measurement

HydC was subjected to the evaluation of the mucoadhesive properties at 37 °C by means of “washability” measurements, carried out using a porcine bladder mucosa as a biological substrate. The bladder mucosa was supplied by a local slaughterhouse under veterinary supervision and used immediately after the dissection. Ialuril^®^ Prefill (IBSA, Italy), a marketed product for IC treatment, was used for the comparison of washability performance.

The measurements were carried out using a modified diffusion Franz cell [25]. The donor chamber was equipped with two arms, to allow a buffer flow, and thermostated at 37 °C. The donor chamber top was closed by a screw, to allow the air leakage during the chamber filling. The receptor and the donor chambers were separated by an impermeable film (Parafilm), used as support for the bladder mucosa. A filter paper disk, wetted with HBSS buffer, was placed between the impermeable film and the mucosa to maintain the mucosa hydrated. The donor chamber was clipped at the receptor chamber. Water was used as a receptor phase to ensure the thermosetting of bladder mucosa. An exact amount of sample (250–300 mg) was loaded using a micro-syringe onto the bladder mucosa through the vent hole. The sample was maintained in contact with the bladder mucosa for 30 or 120 min to allow the interaction with the biological substrate, thus simulating the administration at the empty bladder. At the end of the fixed time, three washing cycles were performed. The donor chamber was filled with HBSS buffer at flow rate of 0.2 mL/min to simulate physiological bladder filling with urine. The system was left at rest for 15 min. Finally, the buffer was collected using the flexible arm in a test tube. This cycle was repeated two more times (3 cycles in total). The test was also carried out in the absence of samples (blank measurements) to assess if the mucosa released interfering components.

##### Active Substances Quantification

Active substances washed away during the washability measurements were quantified by their glucosamine or galactosamine content with an amino sugar assay [26]. Five mL of the collected solutions were lyophilized, re-suspended in 0.5 mL of water, and hydrolyzed in 6 N hydrochloric acid (HCl) for 3 h at reflux. Briefly, 0.1 mL of the sample solutions were transferred in 25 mL volumetric flasks with 10 mL of 6 N HCl, capped with reflux condensers and laid over a thermo block set at 165 °C. After neutralization with 10 mL of 6 N sodium hydroxide solution and water to reach the final volume, 0.2 mL of the solutions was diluted with 0.2 mL of 0.2 M pH 9.3 borate buffer and derivatized with 0.2 mL of a 1 mg/mL FMOC-Cl solution in acetonitrile. The reaction mixture was left stirring up to completion for at least 30′. Then, the samples were analyzed through HPLC-MS using the instrument settings previously reported [26].

The total amount of the products deposited on each bladder mucosa was weighed and expressed as a glucosamine/galactosamine quantity based on the active substances content in HydC or Ialuril^®^ Prefil. For each washing cycle, the amount of the active substances washed away was determined as a glucosamine/galactosamine quantity. The percentage of product still adhering to the mucosa was calculated from the percentage of the active substances washed away against the initial deposited total quantity.

The hydrolysis and derivatization procedures described above were also performed on a HA standard solution (0.05 mL of a 9.30 mg/mL 200 kDa HA solution, Fidia Farmaceutici SpA) and on blank measurement samples spiked with the HA standard solution (0.05 mL of blank sample with 0, 5, 10, 25, 40, or 50 µL of HA standard solution) in order to verify the linearity of the analytical method.

**Figure 1:**
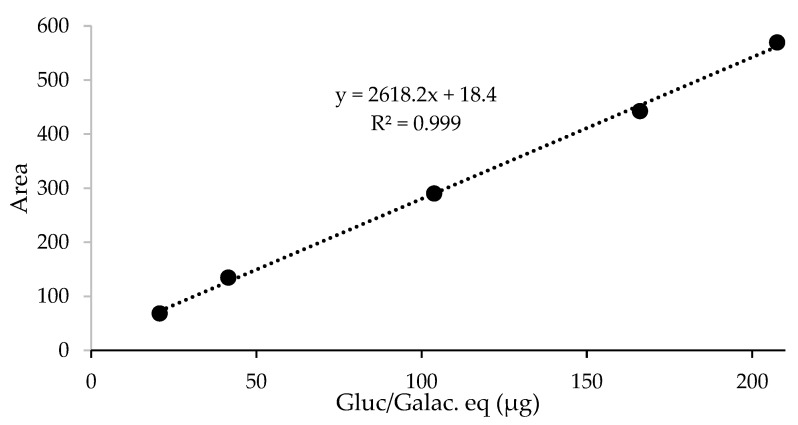
Linearity of response of the analytical method for glucosamine quantitation, obtained after hydrolysis and derivatization of blank samples spiked with HA.

The reliability of the analytical method for the quantification of the analytes released from the test samples (glucosamine from HA and HydC and galactosamine from chondroitin sulfate) was tested on untreated mucosa samples spiked with HA. A linear response with R^2^ > 0.999 (see Figure 1) was observed. Blank samples of untreated, washed mucosa were spiked with HA in the 0.02–0.21 mg range of glucosamine/galactosamine-equivalent content in the hydrolysis step. For accuracy evaluation, the glucosamine/galactosamine content was determined from the linear equation, and it was compared with the spiked theoretical amount: mean recoveries of spiked samples were in the range of 100 ± 10%.

#### 2.2.3. In Vitro Cytotoxicity Assay

Human urothelial cells (line T24, ATCC, HTB-4^TM^) were thawed and maintained in McCoy’s 5A medium containing 10% FBS (Life Technologies, Italy) in an incubator at 37 °C and 5% CO_2_ and 95% relative humidity. The medium was changed every two days, and the cells were subcultured whenever they reached 80% of confluence.

In order to evaluate the biocompatibility of HydC, a quantitative analysis according to the ISO 10993–5:2012 International Standard was conducted. Essentially, urothelial cells were plated at a density of 1 × 10^4^ cells per well in 96-well plates (Sarstedt, Nümbrecht, Germany). After a 24 h incubation at 37 °C and 5% CO_2_, the cells were washed with PBS (Life Technologies, Italy), and solutions of the tested compound were added to reach a final concentration in HA of 3.6, 1.8, 0.9, 0.4, 0.2, 0.1, 0.05, and 0.02 mg/mL (four replicates were tested for each condition). The cells were incubated for 24 h under standard conditions, the medium was then aspirated, and the cells were washed with phosphate buffer solution (PBS, 1X) to remove cell media that could interfere with the fluorimetric method. After that, 100 µL of complete medium containing 10% Alamar Blue (Life Technologies, Milan, Italy) was added to each well, the plate was incubated for 4 h (37 °C and 5% CO_2_), and the fluorescence was measured using a microplate reader (Nanoquant Infinite M200 Pro, Tecan Group Ltd., Zürich, Switzerland) at an excitation wavelength of 530 nm and an emission wavelength of 590 nm. The results were expressed as a cell viability percentage based on the following formula: % cell viability = (RFU solution tested/ RFU control) × 100. (RFU = relative fluorescence units.) Values of cell viability equal or under 70% indicate a cytotoxic effect, as recommended in ISO 10993-5:2012 International Standard. Cells in the complete medium were used as a control, whereas cells treated with 0.5 mM of sodium dodecyl sulfate (SDS) were used as a positive control.

#### 2.2.4. In Vitro Efficacy Assay in an Inflammatory Model

The anti-inflammatory effect of HydC was assessed by means of an in vitro inflammatory model. Briefly, HTB-4 cells were plated at a density of 4 × 10^5^ cells per well in 6-well plates (Sarstedt, Nümbrecht, Germany) and then incubated for 24 h under standard conditions. After 24 h, the medium was aspirated, and the cells were washed with PBS. Therefore, the cells were treated for 24 h as follows: basal medium (serum-free) as a control; TNF-α (Life Technologies, Milan, Italy) at 1 or 10 ng/mL as positive controls; TNF-α (1 ng/mL) and HydC diluted in medium to obtain a final concentration of 0.5 mg/mL in HA; TNF-α (10 ng/mL) and HydC at a final concentration of 0.5 mg/mL in HA. After the treatment, 1 mL of each tested solution was collected and maintained at −20 °C until IL-6 and IL-8 ELISA tests were performed. The level of these two interleukins was measured by ELISA (Diaclone, Besançon, France), and the assays, as well as data analysis, were carried out according to the manufacturing instructions.

#### 2.2.5. Sulfated Glycosaminoglycans Assay

The dimethylmethylene blue (DMMB) assay allows the estimation of the total amount of sulfated glycosaminoglycans (GAGs) in solutions. In this assay, the following conditions were tested: (1) T24 in the McCoy’s 5A basal medium (control); (2) T24 in basal medium +TNF-α 1 ng/mL or 10 ng/mL (positive control); (3) T24 in basal medium +TNF-α (1 ng/mL or 10 ng/mL) in combination with HydC (final HA concentrations: 2 and 0.5 mg/mL). The DMMB solution was prepared as follows: 16 mg of DMMB (Sigma Aldrich, Milan, Italy), 3.04 mg of glycine (Fluka, Milan, Italy), and 1.6 g of NaCl (Sigma Aldrich, Milan, Italy) were weighed and dissolved in 100 mL of 0.1 M acetic acid. Then, a standard solution of chondroitin sulfate (Sigma Aldrich, Milan, Italy) prepared at 10 mg/mL in PBS, and a relative standard curve (80, 40, 20, 10, 5, 2.5, and 0 µg/mL) was prepared. Finally, 20 µL of each standard solution or sample and 180 µL of DMMB solution were added to every single well, and the absorbance at 530 nm was measured immediately using a microplate reader (Nanoquant Infinite M200 Pro, Tecan Group Ltd., Zürich, Switzerland). The quantity of GAGs in each sample was determined by extrapolating the optical density (OD) values against CS standard solutions using the standard curve.

#### 2.2.6. Statistical Analysis

Data from cytokine release and DMMB assay were first investigated with Grubbs’ test for outlier identification and then with one-way ANOVA with Tukey’s post-hoc test, using GraphPad software (GraphPad Prism 9.0 Software, San Diego, California, USA).

Statistical analysis of washability test data was performed using two-way ANOVA with Tukey’s post-hoc test (GraphPad Software, San Diego, CA, USA). Differences were considered significant when *p* < 0.05.

## 3. Results and Discussion

### 3.1. Rheological Analysis and Rheological Synergism

**Figure 2:**
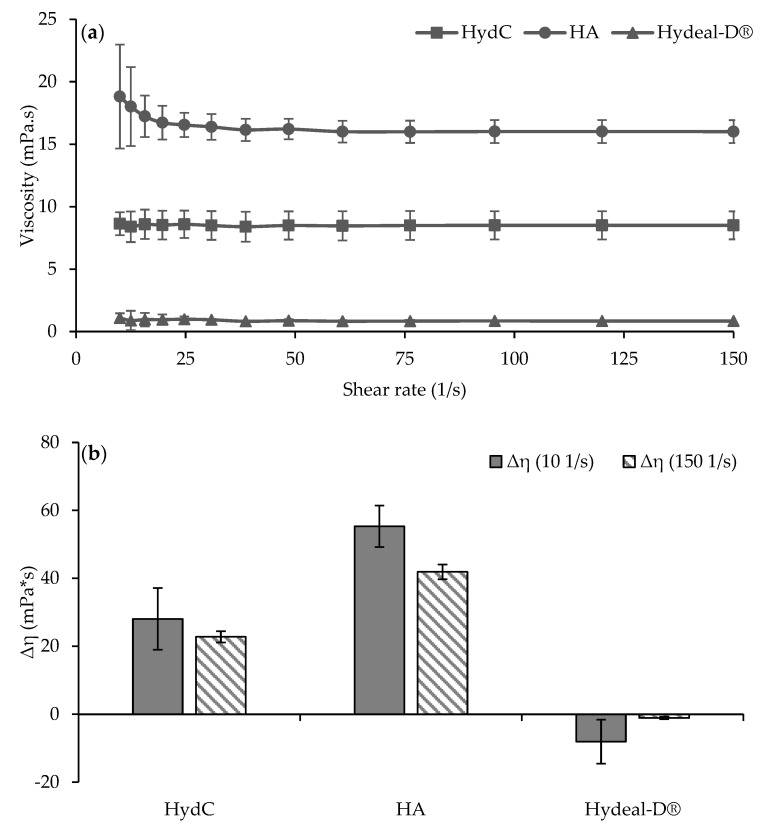
(**a**) Viscosity profiles of HydC, HA, and Hydeal-D^®^ measured from 10 to 300 s^−1^ at 37 °C (mean values ± s.d.; n = 3). (**b**) Values of the rheological synergism (Δη) at 10 to 300 s^−1^ obtained for all the samples after incubation with mucin dispersion (mean values ± s.d.; n = 3).

In Figure 2a, the viscosity profiles of HydC, HA, and Hydeal-D^®^ are reported. HydC was characterized by a Newtonian behavior due to constant viscosity over the investigated shear rate range (HydC: 8.50 mPa.s (sd = 0.07); Hydeal-D^®^: 0.89 mPa.s (sd: 0.08)). Moreover, Hydeal-D^®^ showed a similar profile, although the viscosity values were about 8-fold lower. On the contrary, HA was characterized by a pseudoplastic behavior (HA: max: 18.81 mPa.s (sd: 4.15); min: 16.00 mPa.s (sd:0.091)), and the viscosity values were two-fold higher than that of HydC.

This behavior could be attributed to the formation of HA/Hydeal-D^®^ interactions in HydC. A similar phenomenon has been described in [27]. Likely, the reduction in dynamic viscosity could be due to the ability of short chains from Hydeal-D^®^ to disrupt intermolecular network formation or to the hydrophobic interactions occurring between the benzylic ring and the amidic groups of N-acetyl glucosamine, a monomeric unit of HA.

In Figure 2b, the rheological synergism values (Δη) obtained for all the solutions after incubation with mucin dispersion are reported. Positive values of this parameter indicate an increase in sample viscosity mainly due to non-covalent bonds between the mucoadhesive moiety of the test substances and the glycoprotein chains of mucin. HA and HydC were characterized by a positive Δη value, and HA showed a higher Δη compared to HydC. On the other hand, Hydeal-D^®^, at the concentration and the condition tested, did not show any significant change in viscosity upon incubation with mucin, and the Δη value was close to 0.

HA proved to be the crucial component to form the mucoadhesive joint, providing a positive synergism. This has been attributed to the presence of carboxyl and hydroxyl groups that can hydrogen bond with glycosyl groups on the mucin polymers; moreover, the interactions are facilitated by the flexible conformation of HA [28]. However, the interaction of HA with Hydeal-D^®^ in HydC caused a significant decrease in the rheological synergism, probably due the different steric hindrance originated by the interposition of these two components. Although the interaction between HA and Hydeal-D^®^ caused a decrease in Δη compared to pure HA, this result should be considered positive, suggesting the formation of the mucoadhesive joint also for HydC.

### 3.2. Washability Properties

**Figure 3:**
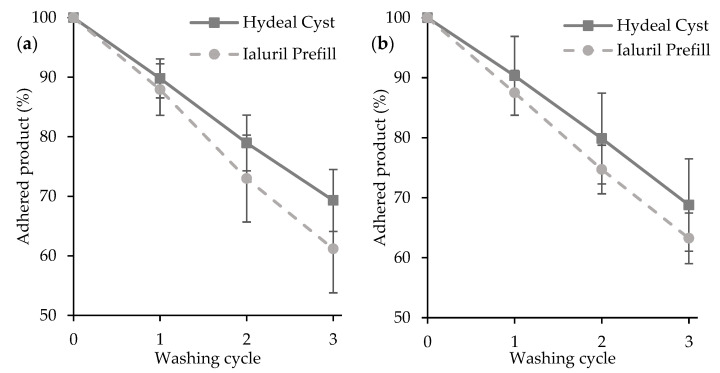
Percentage of the formulations still adhered to the bladder mucosa vs. washing cycle after 30 min (**a**) or 120 min (**b**) of contact (mean values ± sd; n = 6).

The resilience properties of HydC in contact with the bladder mucosa were evaluated considering two different contact times, 30 min or 120 min, before washing cycles (Figure 3). The washability behavior of HydC was compared to that of Ialuril^®^ Prefill (a medical device claimed as a mucoadhesive).

HydC showed good mucoadhesive properties, and the removal action of the buffer stream, simulating micturition, was hindered by the mucoadhesive joint formation that increased the resilience properties of the product in contact with the bladder mucosa. The removal of the formulation was linear over time, and no significant differences were found with different adhesion times (30 or 120 min): about 70% of the main components (HA or Hydeal-D^®^) were still adhering onto the mucosa after three washing cycles.

This is an essential feature since the mucoadhesion phenomenon proved to occur in a shorter time, and this could improve patients’ compliance.

The washability behavior of HydC was also comparable with that of Ialuril^®^ Prefill (a medical device claimed as a mucoadhesive): no significant differences were found in the percentage of product washed away between the two products at each adhesion times and after each washing step.

### 3.3. Cytotoxicity Assay

**Figure 4:**
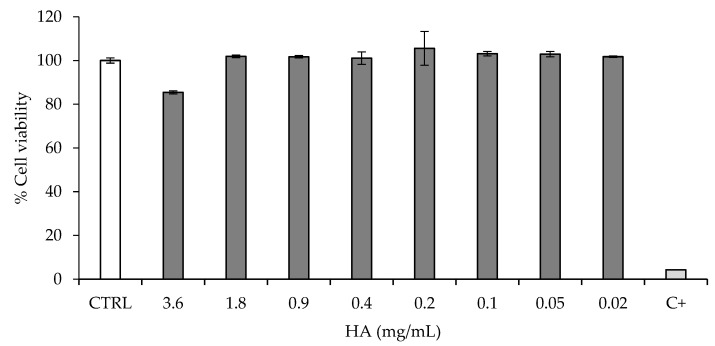
Cytotoxicity of HydC at different concentrations on human urothelial cells (HTB-4) 24 h after treatment. CTRL: HTB-4 in basal medium; C+: SDS (0.5 mM).

Figure 4 reports the viability of urothelial cells (HTB-4) after a 24-h contact with HydC at different concentrations, with HA ranging from 0.02 to 3.6 mg/mL. HTB-4 cells were selected since those are used in the literature to investigate the inflammatory activity of bacterial colonization in epithelial cells [29,30]. The cell viability was always higher than 70% (Figure 4): this indicates that HydC was biocompatible, according to the reference ISO guideline (10993-5:2012, International Standard for the biological evaluation of medical devices).

HydC is intended for intravesical instillation, and the bladder epithelial cells represent the cell type in direct contact with the formulation. Considering the regulatory requirements for medical devices, the in vitro preliminary demonstration of safety towards urothelial cells is mandatory at the preclinical level: these results indicate that the formulation is well tolerated by urothelial cells and is therefore biocompatible.

### 3.4. ELISA Assay Evaluating Cytokines Levels in T24 Supernatants

**Figure 5:**
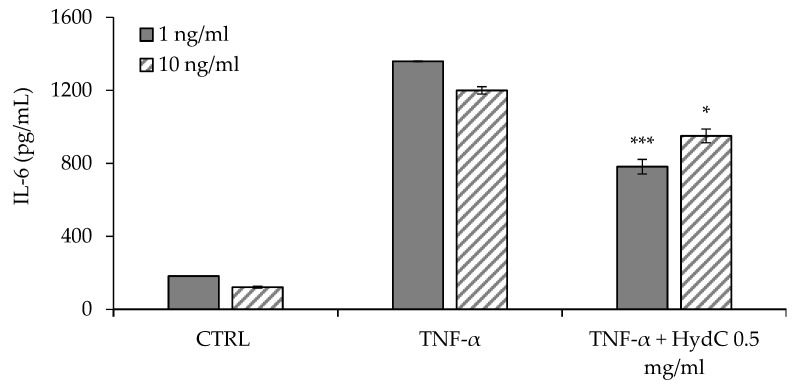
Quantification of IL-6 in HTB-4 cell supernatants after a 24-h stimulation with TNF-α 1 ng/mL (positive control) or TNF-α 1 ng/mL + HydC (HA: 0.5 mg/mL) and with TNF-α 10 ng/mL (positive control) or TNF-α 10 ng/mL + HydC (HA: 0.5 mg/mL) (* *p* < 0.05; *** *p* < 0.0001). CTRL: HTB-4 in basal medium.

**Figure 6:**
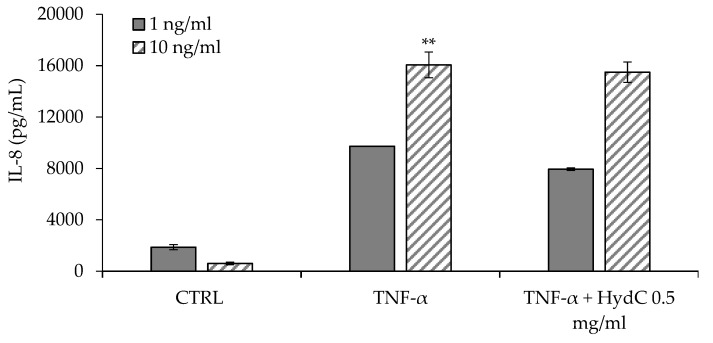
Quantification of IL-8 in HTB-4 cell supernatants after a 24-h stimulation with TNF-α 1 ng/mL (positive control) or TNF-α 1 ng/mL + HydC (HA: 0.5 mg/mL) and with TNF-α 10 ng/mL (positive control) or TNF-α 10 ng/mL + HydC (HA: 0.5 mg/mL) (** *p* < 0.001). CTRL: HTB-4 in basal medium.

Figure 5 and Figure 6 report the concentrations of IL-6 and IL-8, respectively, secreted by urothelial cells (HTB-4) after a 24-h stimulation with two different concentrations of TNF-α (1 or 10 ng/mL). The inflammatory activity of TNF-α seems not to be directly related to its concentration in the range of 1–10 ng/mL, and the consequent secretion of IL-6 was similar independently of the TNF-α concentration.

The anti-inflammatory effect of HydC (final HA concentration: 0.5 mg/mL) was assessed in terms of the reduction in IL-6 and IL-8 levels in TNF-α-stimulated HTB-4 cell supernatants. HydC was able to significantly reduce the amount of IL-6 (−43%) secreted by HTB-4 in response to the lower inflammatory cytokine level tested (TNF-α 1 ng/mL) (Figure 5). In the presence of a higher TNF-α concentration (10 ng/mL), HydC was able to statistically reduce the secretion of IL-6 to a quantitatively lesser extent (−12%, see Figure 5). Moreover, in the moderate inflammatory condition (TNF-α 1 ng/mL), IL-8 secretion also decreased moderately but significantly (−19%) after the treatment with HydC (Figure 6), while a non-significant decrease was observed after a more severe inflammatory stimulus (Figure 6).

To date, specific biomarkers for IC have not yet been identified. IL-6 and IL-8 are increased in IC, although their role is not yet fully clarified. In fact, it is known that their presence in urine [31,32] and serum samples [33] from IC patients is significantly higher compared to those of control patients. The significant reduction in IL-6 and IL-8 levels after treatment with HydC is considered a positive finding and is in agreement with other in vitro studies reported in the literature [18,34].

### 3.5. Secretion of sGAGs by Urothelial Cells

**Figure 7:**
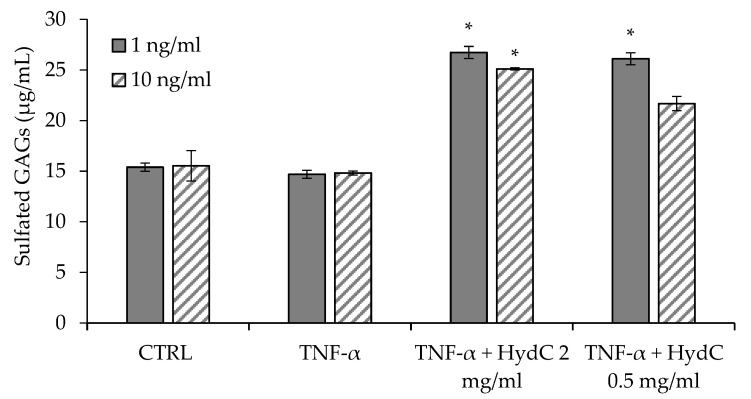
Levels (ng/mL) of sulfated glycosaminoglycans (sGAGs) secreted by HTB-4 cells after a 24-h stimulation with TNF-α at 1 ng/mL and 10 ng/mL, and the treatment with HydC (0.5 and 2 mg/mL) (* *p* < 0.05). CTRL: HTB-4 in basal medium.

Figure 7 reports the concentrations of sulfated glycosaminoglycans (sGAGs) in the cell surnatants secreted by HTB-4 as a consequence of the exposure to inflammatory stimulus caused by TNF-α at 1 ng/mL (a) and 10 ng/mL (b), with or without treatment with HydC (0.5 mg/mL).

The exposure of HTB-4 cells to both 1 and 10 ng/mL TNF-α caused a non-significantly different secretion of sGAGs compared to the control. On the contrary, in the presence of HydC, sGAGs secretion by HTB-4 cells was higher compared to the control and to stimulated cells. After a moderate pro-inflammatory stimulus, both HydC concentrations (0.5 and 2 mg/mL) were able to significantly increase sGAGs secretion. However, only the higher HydC concentration tested was able to increase significantly sGAGs secretion after the cells had been pre-treated with the stronger pro-inflammatory stimulus.

GAGs are a family of linear polysaccharides consisting of amino sugar (N-acetylated or N-sulfated glucosamine, or N-acetylgalactosamine) and uronic acid or galactose. Specifically, dermatan sulfate, keratan sulfate, chondroitin sulfate, heparin, and heparan sulfate are sulfated GAGs, while HA is the only non-sulfated natural polymer of this class. These polysaccharides, alone or coupled with a protein core to form proteoglycans, are present on the cell surface, in the intracellular compartment, and in the extracellular matrix (ECM) and are involved in many biological activities [35]. At the urothelium level, GAGs play a fundamental role by acting as a protective shield against urinary solutes. When the GAG shield becomes thinner, a pathologic condition of urothelium occurs. This is caused by an increased permeability of the epithelial cell layer, leading to inflammation and pain, which are two of the main hallmarks of IC [18]. Weekly intravesical instillations of GAG-containing products represent one of the medical approaches for the treatment of this pathology [31,32,33,34,35,36,37]. Considering this, the enhanced secretions of sGAGs by TNF-α-stimulated urothelial cells following exposure to HydC is an extremely important finding, and it is in agreement with the reported evidence of in vitro HA activity [18].

## 4. Conclusions

HydC (HydealCyst) is a commercial product based on HA and Hydeal-D^®^ for protecting and restoring urothelium in the presence of interstitial cystitis. The in vitro characterization suggests that molecular interactions are formed between HA and Hydeal-D^®^ that have a role in the rheological behavior of the formulation and consequently in the mucoadhesive properties. HA is identified as a key component to form the mucoadhesive joint, and the interaction of HA with Hydeal-D^®^ maintains the mucoadhesive behavior typical of the polysaccharide. Furthermore, in comparison with Ialuril^®^ Prefill, a marketed product for IC treatment, HydC showed comparable mucoadhesive properties in an ex vivo test on porcine bladder mucosa. Moreover, HydC was found to be cytocompatible with urothelial cells (HTB-4) and to possess an anti-inflammatory effect towards them by decreasing the secretion of IL-6 and IL-8, both increased in patients with IC, and by increasing the secretion of sulfated GAGs in vitro. These findings, along with the resilience properties of the formulation due to mucoadhesion, contribute to elucidating the active role of HydC in restoring urothelium homeostasis.

## Data Availability

Data available on request.
